# Action of cholecalciferol and alpha-tocopherol on Staphylococcus aureus efflux pumps

**DOI:** 10.17179/excli2016-277

**Published:** 2016-04-29

**Authors:** Saulo R. Tintino, Cícera D. Morais-Tintino, Fábia F. Campina, Raimundo L. Pereira, Maria do S. Costa, Maria Flaviana B.M. Braga, Paulo W. Limaverde, Jacqueline C. Andrade, José P. Siqueira-Junior, Henrique Douglas Melo Coutinho, Valdir Q. Balbino, Tereza C. Leal-Balbino, Jaime Ribeiro-Filho, Lucindo J. Quintans-Júnior

**Affiliations:** 1 Laboratory of Microbiology and Molecular Biology (LMBM), Department of Biological Chemistry/CCBS/URCA, Brazil; 2 Laboratory of Microrganism Genetics (LGM), Department of Molecular Biology/CCEN/UFPB, Brazil; 3 Laboratory of Bioinformatics and Evolutionary Biology (LABBE), Department of Genetics/ CCB/UFPE, Brazil; 4 Aggeu Magalhães Resaerch Center, CPqAM/Fiocruz, Department of Microbiology, Brazil; 5 Leão Sampaio Universitary Center, Brazil; 6 Department of Physiology/UFS, Brazil

**Keywords:** alpha-tocopherol, cholecalciferol, efflux pumps, Staphylococcus aureus

## Abstract

Alpha-tocopherol is one the most abundant and biologically active isoforms of vitamin E. This compound is a potent antioxidant and one of most studied isoforms of vitamin E. Vitamin D3 (cholecalciferol) is an important nutrient for calcium homeostasis and bone health, that has also been recognized as a potent modulator of the immune response. Methicillin-resistant *Staphylococcus aureus *(MRSA) is the most important causative agent of both nosocomial and community-acquired infections*. *The aim of this study was to evaluate the inhibitory effect of alpha-tocopherol and cholecalciferol on both *S. aureus *and multidrug resistant *S. aureus *efflux pumps. The RN4220 strain has the plasmid pUL5054 that is the carrier of gene that encodes the macrolide resistance protein (an efflux pump) MsrA; the IS-58 strain possesses the TetK tetracycline efflux protein in its genome and the 1199B strain resists to hydrophilic fluoroquinolones *via* a NorA-mediated mechanism. The antibacterial activity was evaluated by determining the Minimal Inhibitory Concentration (MIC) and a possible inhibition of efflux pumps was associated to a reduction of the MIC. In this work we observed that in the presence of the treatments there was a decrease in the MIC for the RN4220 and IS-58 strains, suggesting that the substances presented an inhibitory effect on the efflux pumps of these strains. Significant efforts have been done to identify efflux pump inhibitors (EPIs) from natural sources and, therefore, the antibacterial properties of cholecalciferol and alpha-tocopherol might be attributed to a direct effect on the bacterial cell depending on their amphipathic structure.

## Introduction

Alpha-tocopherol is one of the most abundant and biologically active isoforms of vitamin E. This compound is a potent antioxidant and one of most studied isoforms of vitamin E (Márquez et al., 2002[31]; Catania et al., 2009[7]).Vitamin E is a liposoluble substance that is able to slow aging and protect organisms from non-transmissible chronic diseases, such as Parkinson's, Alzheimer's, infectious and rheumatic states, cancer and cardiovascular diseases (Catania et al., 2009[7]; Batista et al., 2007[3]; Berg, 2010[4]). Recent findings demonstrated that vitamin E is able to inhibit the growth of malignant cells (Sampaio and Almeida, 2009[44]) and modulate cell signaling and gene transcription (Batista et al., 2007[3]). Vitamin E (Alpha-tocopherol) is naturally present in many products of both plant and animal origin, such as: vegetable oils, wheat germ, oily seeds, dark-red vegetables, egg yolk and in the liver. 

Vitamin D3, or cholecalciferol, and vitamin D2, or ergocalciferol, are two different forms of vitamin D obtained by humans. Ergocalciferol is produced by many plants, yeasts and fungi when they are exposed to UVB radiation and cholecalciferol is synthesized in the skin by exposure to UVB radiation (Bikle, 2009[5]). Vitamin D has been recognized as a potent modulator of the immune response and as an important nutrient for calcium homeostasis (Holick, 2004[24]; Heaney, 2005[22]; Miller and Gallo, 2010[34]). The main circulating form of this vitamin in the organism is the 25-hydroxyvitamin D (25[OH]D) (calcidiol), which requires activation by the renal 1 α-hydroxylase to form the metabolically active 1,25-dihydroxvitamin D (1,25[OH]2D) (calcitriol) (DeLuca, 2004[13]).

Liposoluble compounds are known as modifiers of the bacterial cell membrane permeability (Pretto et al., 2004[40]; Nicolson et al., 1999[37]). Therefore, alpha-tocopherol and cholecalciferol due to their liposolubility might increase the permeability of the bacterial cell membrane to various substances, including antibiotics (Andrade et al., 2014[2]).

Beyond the intrinsic resistance properties to certain antibiotics, bacteria can also acquire resistance to antibiotics via horizontal gene transfer and mutations in chromosomal genes. The intrinsic resistance of a bacterial species to a particular antibiotic is its ability to resist to the action of this antibiotic as a result of inherent structural or functional characteristics, resulting on antibiotic inactivation (Blair et al., 2015[6]). Accordingly, there has been increasing concern about bacterial resistance to antibiotics, specially because of the availability and inappropriate use of these drugs (Neuhauser et al., 2003[36]; Sahm et al., 2001[42]). In fact, numerous bacterial strains, especially the methicillin-resistant variety, rapidly became resistant to antibacterial agents after the introduction of antibiotics for treatment of serious infections caused by *S. aureus* (Harnett et al., 1991[20]; Mesak and Davies, 2009[33]).

Methicillin-resistant *Staphylococcus aureus* (MRSA) is the most important causative agent of both community-acquired and nosocomial bacterial infections. *S. aureus* cause these infections through various virulence factors (Costa et al., 2011[10]; Havaei et al., 2010[21]). MRSA strains present resistance to many antibacterial agents, limiting the treatment of many patients infected with these bacteria (Kurlenda and Grinholc, 2012[26]). There are three different mechanisms of antibiotic resistance to *S. aureus*: inactivation of the antibiotic by hydrolysis or chemical modification; modification of the antibiotic target by genetic mutation or post-translational mechanisms; and reduction of the intracellular concentrations of the antibiotic as a result of deficient penetration into the bacterium or antibiotic efflux by active mechanisms (Blair et al., 2015[6]).

The active efflux of substances that inhibit bacterial growth such as toxic compounds and antibiotics, in *S. aureus*, is mediated by integral membrane transporters, known as drug efflux pumps (Levy, 1992[27]). There are several categories of active drug efflux pumps that transport drugs against their concentration gradients across the membrane (Levy, 1992[27]). In the *S. aureus* species, the following efflux proteins are found: QacA (Small multidrug resistance protein from the SMR family) (Paulsen et al., 1996[38]; Saier et al., 1994[43]), Smr (SMR family) (Grinius et al., 1992[19]), Tetk (Major facilitator superfamily from the MFS family), NorA (MFS family) (Yoshida et al., 1990[49]) and MsrA (MFS family) (Reynolds et al., 2003[41]).

Several studies have demonstrated the development of antibiotic resistance in pathogenic bacteria during the course of antibiotic treatment which involved efflux pumps (Levy, 2005[29]; Neu, 1992[35]; Levy, 2002[28]). Therefore, it is hypothesized that the antibiotic therapy can be effective if: (i) efflux pumps are inhibited, (ii) the expression of efflux pumps is downregulated, or (iii) the antibiotics are redesigned, so that they are no longer suitable to efflux substrates and thus, their clinical efficacy is restored (Kourtesi et al., 2013[25]).

The aim of this study was to evaluate the inhibitory effect of alpha-tocopherol and cholecalciferol on the *Staphylococcus aureus *efflux pumps. 

## Materials and Methods

### Bacterial strains 

The following strains of *S. aureus *were used: RN4220, which has the plasmid pUL5054 that is the carrier of gene that encodes the macrolide resistance protein (an efflux pump) MsrA; IS-58, which possesses the TetK tetracycline efflux protein in its genome and 1199B, which resists to hydrophilic fluoroquinolones via a NorA-mediated mechanism. All strains used in this work were kindly provided by Prof. S. Gibbons (University of London). These strains were maintained in blood agar base culture medium slants (Laboratorios Difco Ltda., Brazil) and, before the assays, the cells were kept grown overnight at 37 °C in Heart Infusion Agar culture medium slants (HIA, Difco) during 24 hours.

### Drugs 

The antibiotics were dissolved in dimethyl sulfoxide (DMSO) and then, diluted in sterile water to the concentration of 1024 μg/mL). Erythromycin, norfloxacin and tetracycline were used as antibiotics. Alpha-tocopherol and cholecalciferol were obtained from Sigma Chemical Co. (St. Louis, USA). Stock solutions of the vitamins were prepared in 1 mL of DMSO to obtain the concentration of 200 mg/ml and then, this solutions were diluted to 1024 µg/mL in distilled water to reduce the DMSO toxicity.

### Antibacterial activity test by Minimal Inhibitory Concentration (MIC)

The MICs of alpha-tocopherol and cholecalciferol were determined in a microdilution assay utilizing an inoculum of 100 μL of each strain suspended in saline solution at 0.5 of the McFarland scale, was added in brain heart infusion (BHI) in Eppendorfs. Then, each sample was transferred to 96-well microtiter plates and serial dilutions of each substance were performed with concentrations ranging from 0.5 to 512 µg/mL. The plates were incubated at 37 °C during 24 h, and bacterial growth was determined using the sodium Resazurin colorimetric method. The MIC was defined as the lowest concentration in which no bacterial growth was observed, according to CLSI (2013[9]). The antibacterial assays were performed in triplicate and the results were expressed as average of the replicates.

### Evaluation of the inhibition of efflux pumps by reduction of MIC

To analyze whether alpha-tocopherol and cholecalciferol might affect the efflux pump activity, we evaluated the potential of these substances to decrease the MIC of the antibiotics. The inhibition of the efflux pump was evaluated using sub-inhibitory concentrations of alpha-tocopherol and cholecalciferol (MIC/8). A 100 μL sample of a solution containing inoculums, suspended in saline solution at 0.5 of the McFarland scale was added in brain heart infusion (BHI) in Eppendorfs. Then, each sample was transferred to 96-well microtiter plates and serial dilutions of each substance were performed with concentrations ranging from 0.5 to 512 µg/mL. The plates were incubated at 37 °C during 24 h, and bacterial growth was determined using the sodium Resazurin colorimetric method. The antibiotic MICs were used as controls. The antibacterial assays were performed in triplicate and the results were expressed as average of the replicates.

### Statistical analysis 

All experiments were made in triplicate. The data were analyzed using two-way ANOVA and the Tukey´s post test using *GraphPad Prism* software 5.0 (GraphPad, San Diego, CA). The values are expressed as geometric means and the differences with p ˂ 0.05 were considered signiﬁcant. 

## Results and Discussion

**Figure 1:**
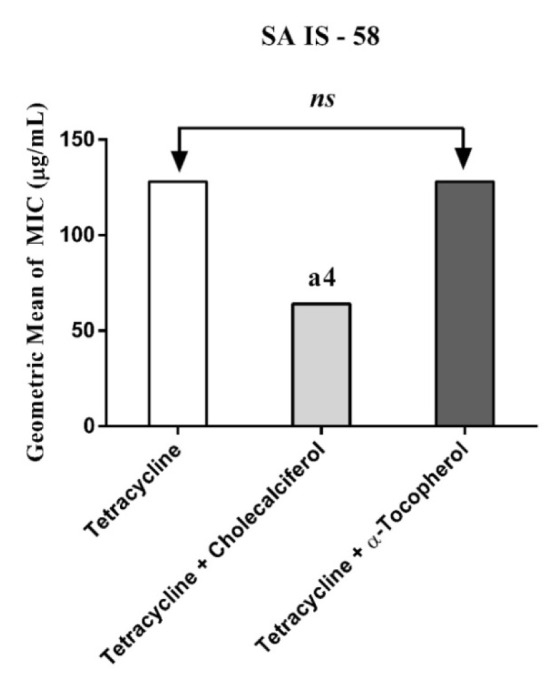
MIC of Tetracycline alone and in association with the standard vitamins against the strain *S. aureus* IS-58, expressing the efflux system TetK. One Way ANOVA, followed by the test Tukey. ^a4^p < 0,0001 *vs* Tetracycline; *ns - *not significant

**Figure 2:**
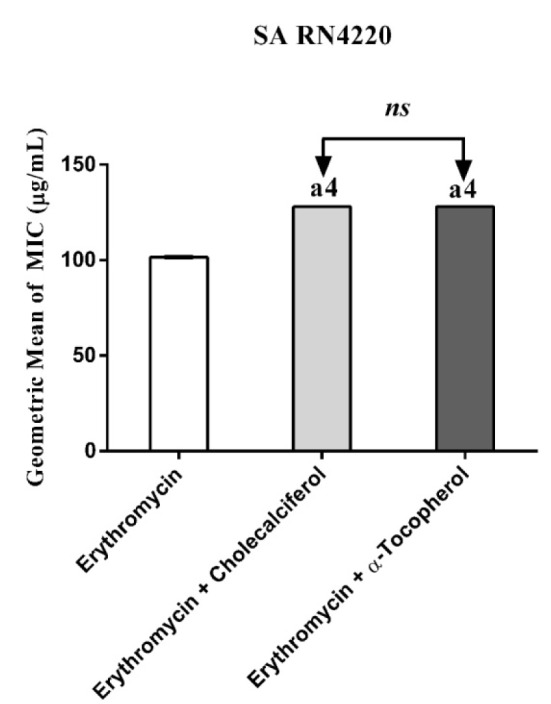
MIC of Erythromycin alone and in association with the standard vitamins against the strain *S. aureus* RN4220, expressing the efflux system TetK. One Way ANOVA, followed by the test Tukey. ^a4^p < 0,0001 *vs* Erythromycin; *ns - *not significant

**Figure 3:**
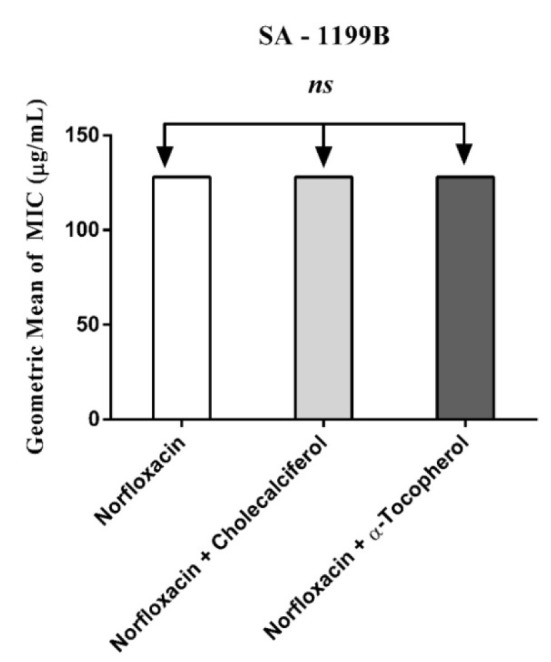
MIC of Norfloxacin alone and in association with the standard vitamins against the strain *S. aureus* 1199B, expressing the efflux system TetK. One Way ANOVA, followed by the test Tukey. *ns - *not significant

**Figure 4:**
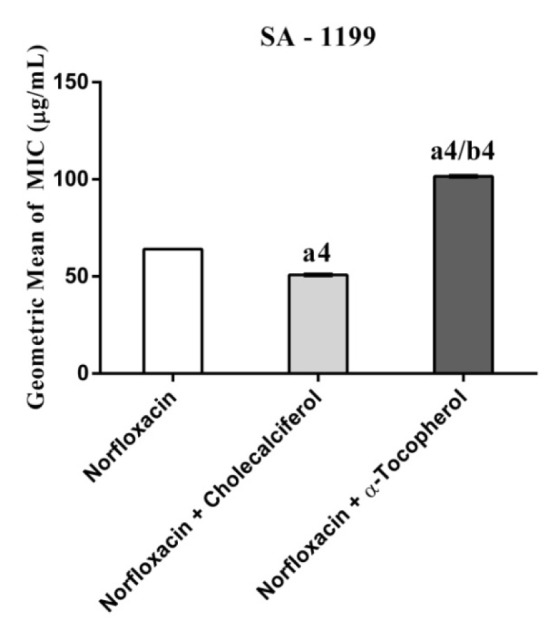
MIC of Norfloxacin alone and in association with the standard vitamins against the strain *S. aureus* 1199B wild, expressing the efflux system TetK. One Way ANOVA, followed by the test Tukey. ^a4^p < 0,0001 *vs* Norfloxacin; ^b4^p < 0,0001 *vs* Norfloxacin.

Cholecalciferal and alpha-tocopherol presented a MIC ≥ 1024 µg/mL and, as such, they do not exhibit clinically relevant antibacterial activity. However, when associated with antibiotics against resistant strains carrying efflux pumps, cholecalciferol reduced the MIC values of the antibiotics (Figures 1-4(Fig. 1)(Fig. 2)(Fig. 3)(Fig. 4)).

Substances that reverse bacterial resistance when associated with antibiotics by reducing their MIC are defined as "modifiers of the antibiotic activity", which can alter the bacterial susceptibility to antibiotics by inhibiting microbial efflux pumps (Costa et al., 2008[11]). Thus, if a substance causes a reduction in the MIC ≥ 3 dilutions when combined with the inhibitor, this is an indicative that this substance affects the bacterial resistance by inhibiting the efflux pump activity (Davies and Wright, 1997[12]).

The association of antibiotics with liposoluble vitamins is an interesting alternative to enhance the antimicrobial efficacy of these drugs, because these vitamins are commonly present in the human feed and present no significant toxicity when used at low concentrations (DiPalma and Ritchie, 1977[14]). This is the first study demonstrating the effect of cholecalciferol and alpha-tocopherol as inhibitors of antibiotic efflux systems. Andrade et al. (2014[2]) were the first researchers to demonstrate the antibiotic modulatory activity against aminoglycosides using a multidrug resistant (MDR) strain of *Staphylococcus aureus*, although several reports previously demonstrated the antibiotic modulator effect of many non-polar compounds against MDR strains.

Falcão-Silva et al. (2009[15]) demonstrated that the amphipathic kaempferol glycoside tiliroside increased the action antibiotics by reducing the concentration needed to inhibit the growth of the RN4220 and IS-58 strains. One of the factors that might be associated to this inhibition is the lipophilicity of the flavon moiety of tiliroside. Lipophilicity is a common feature of several efflux pump inhibitors (EPIs), and this feature, as pointed out by Gibbons (2004[16]), is probably important for EPIs solubility in the bacterial membrane and binding to the efflux pump or efflux pump substrates. Thus, the lipophilicity of a given compound may be determinant on inhibiting the efflux pump in Gram-positive multi-resistant bacteria (Stavri et al., 2007[47]). Accordingly, several reports have demonstrated that lipophilic compounds act as inhibitors of drug efflux pumps in cancer cells (Werle, 2008[48]). Finally, other amphipathic compounds such as piperidine alkaloids (Gibbons, 2005[17]), acylated oligosaccharides from the orizabin series (Pereda-Miranda et al., 2006[39]) and from the murucoidin series, stoloniferin I (Chérigo et al., 2008[8]) and the phenolic diterpene totarol (Smith et al., 2007[46]) have been reported as EPIs against the SA-1199B strain. However, other factors, including special structural features may be crucial for the action of drugs on efflux pump inhibition.

In fact, the lipophilicity of some substances, including alpha-tocopherol and cholecalciferol can alter the structure of the bacterial lipoprotein membrane, resulting on damage to components that are essential for the membrane integrity, and therefore, efflux pumps and other membrane transporters may be significantly affected by these substances (Gibbons, 2004[16]). The membrane potential can also be affected leading to loss of ions, cytochrome C, proteins and radicals and finally, collapse of proton pumps system and decrease in the intracellular ATP (Sikkema et al., 1994[45]; Hirayama et al., 2006[23]).

Although the studies demonstrating the effect of the combination of antibiotics whit cholecalciferol remain to be developed, a recent study demonstrated the effect of this substance as a modulator of the inflammatory response on bovine mammary epithelial cells (Alva-Murillo et al., 2014[1]). Interestingly, prior to the development of the antibiotics, sunlight (a source of vitamin D), cod liver oil, and pharmacologic doses of liposoluble vitamin D were used to treat tuberculosis (Martineau et al., 2007[32]). This type of treatment fell out of favor following the creation of effective antibiotic therapy. However, the interest in using pharmacologic doses of vitamin D increased recently in view of the finding that this vitamin can stimulate innate immune responses to combat several pathogenic infections *in vitro.* Thus, vitamin D can act as an "antibiotic", in part, by inducing the transcription of human antimicrobial peptide genes (Liu et al., 2006[30]; Gombart et al., 2005[18]; Zasloff, 2006[50]). By this way, the potential use of cholecalciferol in association with antibiotics can represent an important weapon in the war against the MDR bacteria. 

## Conclusion

Cholecalciferal and alpha-tocopherol presented different patterns of antibiotic modulatory activity. Cholecalciferol presented a significant modulatory effect on the tetracycline activity against the IS-58 *S. aureus* strain, indicating that this vitamin enhanced the antibiotic activity by affecting the bacterial of efflux systems. Together, our data suggest that the vitamins act through specific mechanisms that are dependent on the bacterial strain and the vitamin molecular structure and therefore, cholecalciferol can be used in the development of new drugs to treat infections caused by *S. aureus* resistant strains.

## Conflict of interest

The authors declare no conflict of interest.
